# Testing a generalized leaf mass estimation method for diverse tree species and climates of the continental United States

**DOI:** 10.1002/eap.2646

**Published:** 2022-06-16

**Authors:** Garret T. Dettmann, David W. MacFarlane, Philip J. Radtke, Aaron R. Weiskittel, David L. R. Affleck, Krishna P. Poudel, James Westfall

**Affiliations:** ^1^ Virginia Tech, Forest Resources and Environmental Conservation Blacksburg Virginia USA; ^2^ Department of Forestry Michigan State University East Lansing Michigan USA; ^3^ Center for Research on Sustainable Forests University of Maine Orono Maine USA; ^4^ WA Franke College of Forestry and Conservation University of Montana Missoula Montana USA; ^5^ Department of Forestry Mississippi State University Mississippi State Mississippi USA; ^6^ USDA Forest Service, Northern Research Station Newtown Square Pennsylvania USA

**Keywords:** allometry, biomass, foliage mass, national forest inventory, species functional traits

## Abstract

Estimating tree leaf biomass can be challenging in applications where predictions for multiple tree species is required. This is especially evident where there is limited or no data available for some of the species of interest. Here we use an extensive national database of observations (61 species, 3628 trees) and formulate models of varying complexity, ranging from a simple model with diameter at breast height (DBH) as the only predictor to more complex models with up to 8 predictors (DBH, leaf longevity, live crown ratio, wood specific gravity, shade tolerance, mean annual temperature, and mean annual precipitation), to estimate tree leaf biomass for any species across the continental United States. The most complex with all eight predictors was the best and explained 74%–86% of the variation in leaf mass. Consideration was given to the difficulty of measuring all of these predictor variables for model application, but many are easily obtained or already widely collected. Because most of the model variables are independent of species and key species‐level variables are available from published values, our results show that leaf biomass can be estimated for new species not included in the data used to fit the model. The latter assertion was evaluated using a novel “leave‐one‐species‐out” cross‐validation approach, which showed that our chosen model performs similarly for species used to calibrate the model, as well as those not used to develop it. The models exhibited a strong bias toward overestimation for a relatively small subset of the trees. Despite these limitations, the models presented here can provide leaf biomass estimates for multiple species over large spatial scales and can be applied to new species or species with limited leaf biomass data available.

## INTRODUCTION

Forests play an important role in the function of global ecosystems and climate (Bonan, 2008), serving as important global CO_2_ sinks (Luyssaert et al., 2008; Wellbrock et al., 2017). A key functional attribute of forests are the leaves themselves, which integrate many ecosystem processes (Atkinson et al., 2010; Wright et al., 2004). For example, leaves are a key component of the global carbon cycle, as more than one‐third of atmospheric CO_2_ passes through leaf stomata and about half of that is fixed through photosynthesis annually (Sitch et al., 2003). Forest canopies have important local effects on climate by modulating the land–atmosphere fluxes of energy and water (Alkama & Cescatti, 2016). Leaf litter from forests plays an important role in nutrient cycling (Cornwell et al., 2008) and many other ecosystem processes (Chapin et al., 2011). Thus, there are many reasons to improve the quality and quantity of information describing the foliage component of forest ecosystems.

Leaf mass can provide information on plant investment in leaf tissue, which has been shown to correlate with woody plant functional types (Duursma & Falster, 2016). Leaf mass is also related to other plant dimensions of interest, such as specific leaf area, which is correlated with the Huber value (xylem sapwood area/leaf area) (Mencuccini, Rosas, et al., 2019), a parameter that can be used in modeling plant water use (Mencuccini, Manzoni, et al., 2019). Leaf mass and leaf‐mass‐based traits have been shown to be critically important, integrative measures of leaf performance and function (Atkinson et al., 2010; Reich et al., 1997), though these traits vary over multiple orders of magnitude across a worldwide spectrum of species and ecosystems (Wright et al., 2004).

The great variation in leaf mass between trees and across forest ecosystems is echoed in calls to develop better estimators of tree leaf biomass to improve forest ecosystem inventories, especially at large spatial scales (Clough et al., 2016; Weiskittel et al., 2015). However, recent approaches to creating generalized tree mass models, that is, “global” allometric models, have focused largely on total tree mass (Chave et al., 2014; Jucker et al., 2017). Though some remote sensing approaches, like terrestrial laser scanning (TLS), are promising to improve biomass estimation (Stovall et al., 2018), estimating foliage biomass with TLS or other methods poses challenges (Stovall et al., 2017).

One of the biggest challenges in improving leaf mass estimation at large spatial scales has been acquiring sufficient data to calibrate species‐specific models (Clough et al., 2016; Poudel & Temesgen, 2016). This problem has been addressed by combining species into broad taxonomic groups (Chojnacky et al., 2013; Jenkins et al., 2003), but these groupings ignore basic differences between species within taxonomic groups (Clough et al., 2016). A more promising approach might be to group species by leaf functional attributes that relate to their life‐history (Reich et al., 2014; Wright et al., 2004).

Building on the idea of a spectrum of leaf traits (Reich et al., 1997; Wright et al., 2004), Dettmann and MacFarlane (2019) developed a “trans‐species” leaf biomass estimation model using two numerically quantifiable leaf traits (shade tolerance and leaf longevity) to represent any species on a numeric scale; however, they found that tree size, vigor, and local competitive environment, not species traits, were the dominant predictors of tree leaf mass. The resultant general leaf mass estimation model gave very good estimates of leaf mass for trees of 17 different tree species (11 hardwoods and 6 softwoods), selected to maximize life history trait differences between species at 21 locations across the US state of Michigan. To show that this model is actually “transferable” across species (sensu Wenger & Olden, 2012), this approach needs further testing over a wider range of tree species, forest ecosystems, and climates for the purpose of assessing general behavior and robustness. For the purposes of this paper, we use the term “trans‐species” from Dettmann and MacFarlane (2019) to refer to the transferability of the model across species.

In this study, we used an extensive nationally derived data set to examine the generality of leaf mass estimation relationships identified by Dettmann and MacFarlane (2019) over diverse tree species, regions, and climates of the United States and to determine the feasibility of its use to estimate leaf biomass for the USDA Forest Service Forest Inventory and Analysis (FIA) program, which conducts the national forest inventory (NFI) and the associated parts of the US greenhouse gas inventory. The new study includes additional climate data, as well as variables from the original model of Dettmann and MacFarlane (2019), and gives full consideration to the feasibility of acquiring the data necessary to test and apply the model over a nation as large and geographically diverse as the US NFI. The specific objectives of this study were to (1) compile and summarize available national tree foliage biomass data for the United States, (2) fit and evaluate trans‐species models of varying complexity, (3) examine developed relationships across a variety of contrasting factors, and (4) assess model robustness and species transferability using a novel “leave‐one‐species‐out” cross‐validation approach.

## MATERIALS AND METHODS

### Study area and data

The leaf biomass data collected in Michigan to generate the original model by Dettmann and MacFarlane (2019) were part of a larger study conducted by seven US universities in partnership with FIA from 2011 to 2021 to improve the US forest biomass inventory (Weiskittel et al., 2015) using detailed measurements of tree aboveground biomass components, including foliage mass. Sampling of field data was focused on species that make up roughly two thirds of the growing stock in the United States and to fill gaps in existing data (Frank et al., 2019). These newly collected data were combined with existing individual tree biomass observations obtained from destructive sampling records from past North American studies spanning 1960 to the present (Baldwin, 1989; Baldwin & Saucier, 1983; Clark, 1977; Clark et al., 1985, 1986a, 1986b; Clark III & Schroeder, 1985; Lohrey, 1984; McNab & Clark, 1982; Mroz et al., 1985; Phillips & McNab, 1982; Radtke et al., 2015). These historical tree biomass studies from across the nation are now available online (http://www.legacytreedata.org/). When collating such a large data set, care was taken to examine metadata so as not to include trees that were not fully leafed out. Notably, not every one of these studies followed the same exact protocol, and as such, differences in data may be a source of statistical noise, error, and outliers (discussed later).

**FIGURE 1:**
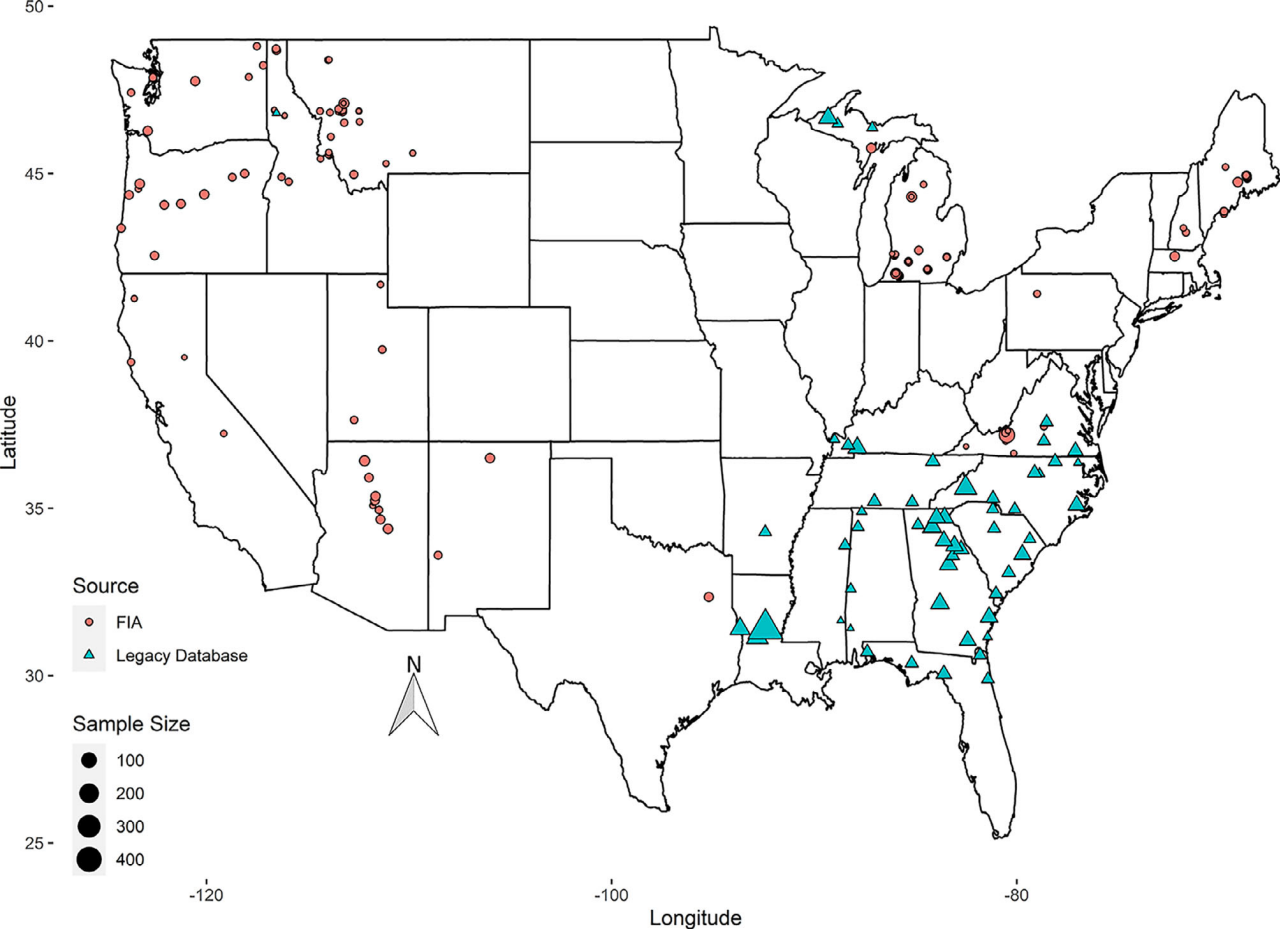
Sample site locations within continental United States of all 3628 sample trees used.

Species represented in the combined data set covered a wide range of shade tolerance, leaf longevity, stem diameter at breast height (DBH) (in centimeters, 1.37 m above ground) (Table 1). Study sites were located across the United States covering a range of climatic conditions and including 61 different tree species (Figure 1). Collectively, the data set represents a diverse set of tree species and a wide variety of life‐history traits (Table 2), growing over a wide range of ecological and climatic regions (Figure 1), providing a robust data set to test the broader applicability of the trans‐species approach of Dettmann and MacFarlane (2019).

**TABLE 1:** Summary of tree data used in model development and testing

Statistic	Mean	SD	Minimum	Maximum
Overall (species = 61; *N* = 3628)				
Dry leaf mass (kg)	11.97	17.4	0.06	203.59
DBH (cm)	23.01	14.5	3.05	114.05
LCR (unitless)	0.52	0.18	0.1	1
Wood specific gravity (observed; unitless)	0.49	0.09	0.22	0.84
Wood specific gravity (published values; unitless)	0.48	0.09	0.29	0.67
Shade tolerance (unitless, ordinal)	2.6	1	0.87	5.01
Leaf longevity (month)	24.4	24.13	4.6	110
Mean annual temperature (°C)	13.29	5.25	−0.4	20.7
Mean annual precipitation (mm)	1186.61	338.52	275	3084
Softwoods (species = 25; *N* = 1951)				
Dry leaf mass (kg)	14.19	20.34	0.08	203.59
Diameter at breast height (DBH) (cm)	21.98	14.66	3.05	114.05
Live crown ratio (LCR) (unitless)	0.52	0.2	0.1	1
Wood specific gravity (observed; unitless)	0.44	0.07	0.22	0.84
Wood specific gravity (published values; unitless)	0.45	0.08	0.29	0.54
Shade tolerance (unitless, ordinal)	2.6	1.07	0.87	5.01
Leaf longevity (month)	40.14	23.33	5.5	110
Mean annual temperature (°C)	13.16	5.76	−0.4	19.7
Mean annual precipitation (mm)	1200.96	408.02	275	3084
Hardwoods (species = 36; *N* = 1677)				
Dry leaf mass (kg)	9.4	12.7	0.06	91.04
DBH (cm)	24.21	14.22	3.05	87.63
LCR (unitless)	0.52	0.14	0.14	0.96
Wood specific gravity (observed; unitless)	0.54	0.09	0.25	0.76
Wood specific gravity (published values; unitless)	0.52	0.1	0.32	0.67
Shade tolerance (unitless, ordinal)	2.6	0.92	1.21	4.87
Leaf longevity (month)	6.08	1.41	4.6	42
Mean annual temperature (°C)	13.44	4.58	1.5	20.7
Mean annual precipitation (mm)	1169.91	231.92	339	1584

**TABLE 2:** Summary of tree attributes and sample sizes by species; means and ranges provided with SD in parentheses

Species	*N*	Diameter at breast height (DBH) (cm)	Leaf mass (kg)	Specific gravity (observed)	Live crown ratio	Leaf longevity (month)	Shade tolerance	Specific gravity (published)
Softwoods (species = 25; *N* = 1951)								
*Abies balsamea*	50	17.47 (8.79) 3.3–40.64	9.09 (8.23) 0.52–28.83	0.35 (0.04) 0.26–0.49	0.62 (0.19) 0.28–0.99	110	5.01	0.33
*Abies concolor*	36	29.61 (14.4) 3.05–66.55	32.12 (29.03) 0.56–108.19	0.39 (0.08) 0.22–0.61	0.71 (0.14) 0.36–0.88	90	4.33	0.37
*Abies grandis*	20	40.13 (20.19) 12.45–84.33	79.74 (64.25) 10.8–203.59	0.39 (0.07) 0.3–0.66	0.79 (0.14) 0.43–0.97	88.8	4.01	0.35
*Abies lasiocarpa*	28	20.68 (8.08) 9.91–37.08	21.32 (16.99) 2.36–54.49	0.35 (0.04) 0.28–0.44	0.88 (0.13) 0.54–1	96	4.83	0.31
*Juniperus virginiana*	2	14.22 (5.03) 10.67–17.78	6.71 (5.2) 3.04–10.39	0.48 (0.06) 0.44–0.52	0.59 (0.04) 0.56–0.62	36	1.28	0.44
*Larix laricina*	6	16.64 (8.53) 5.33–28.19	8.76 (7.76) 0.71–21.69	0.52 (0.03) 0.49–0.57	0.82 (0.09) 0.68–0.94	5.5	0.98	0.49
*Larix occidentalis*	24	31.86 (12.66) 9.14–60.71	8.85 (6.86) 1–23.22	0.48 (0.05) 0.41–0.58	0.65 (0.11) 0.44–0.92	6	1.35	0.48
*Picea engelmannii*	51	23.33 (12.07) 4.32–51.56	31.31 (32.09) 0.91–125.24	0.35 (0.05) 0.26–0.46	0.86 (0.1) 0.57–1	90	4.53	0.33
*Picea glauca*	2	31.24 (2.87) 29.21–33.27	17.06 (3.27) 14.75–19.37	0.38 (0) 0.37–0.38	0.53 (0.1) 0.46–0.61	55	4.15	0.33
*Pinus contorta*	61	22.7 (8.54) 7.37–42.93	11.77 (8.95) 0.86–39.46	0.45 (0.07) 0.22–0.76	0.7 (0.19) 0.3–0.96	60	1.73	0.38
*Pinus echinata*	63	9.93 (3.78) 5.08–33.27	1.76 (2.06) 0.48–15.65	0.48 (0.05) 0.4–0.6	0.42 (0.12) 0.22–0.65	42	1.86	0.47
*Pinus elliottii*	540	16.65 (8.13) 3.3–48.51	8.06 (9.49) 0.16–75.8	0.49 (0.04) 0.4–0.62	0.36 (0.1) 0.11–0.79	24	2.65	0.54
*Pinus glabra*	1	13.21 (NA) 13.21–13.21	2.86 (NA) 2.86–2.86	0.44 (NA) 0.44–0.44	0.56 (NA) 0.56–0.56	30	4.5	0.41
*Pinus palustris*	150	19.78 (12.49) 3.3–52.32	11.39 (14.36) 0.47–86.89	0.51 (0.04) 0.41–0.59	0.46 (0.12) 0.19–0.81	32	0.87	0.54
*Pinus ponderosa*	135	30.93 (12.7) 4.32–57.66	23.14 (18.92) 0.08–82.24	0.41 (0.07) 0.22–0.84	0.58 (0.14) 0.16–0.87	36	1.64	0.38
*Pinus resinosa*	13	35.66 (20.26) 5.33–68.07	22.26 (24.52) 0.77–72.36	0.39 (0.02) 0.35–0.42	0.63 (0.2) 0.26–0.86	36	1.89	0.41
*Pinus rigida*	31	17.6 (11.69) 4.06–44.2	8.74 (10.99) 0.15–38.92	0.42 (0.04) 0.36–0.51	0.6 (0.14) 0.32–0.86	33	1.99	0.47
*Pinus strobus*	137	26.23 (18.67) 3.05–81.28	11.81 (18.71) 0.36–107.05	0.34 (0.03) 0.27–0.49	0.56 (0.16) 0.19–0.92	30	3.21	0.34
*Pinus taeda*	242	19.01 (11.48) 3.05–52.83	9.99 (12.42) 0.45–73.61	0.46 (0.05) 0.35–0.63	0.42 (0.17) 0.13–0.91	21.1	1.99	0.47
*Pinus virginiana*	84	11.47 (7.75) 3.3–42.42	3.86 (4.65) 0.47–36.38	0.45 (0.04) 0.36–0.56	0.63 (0.2) 0.24–0.95	42	1.99	0.45
*Pseudotsuga menziesii*	152	36.79 (20.41) 4.06–114.05	28.11 (23.79) 0.44–126.44	0.45 (0.07) 0.25–0.84	0.64 (0.18) 0.1–0.99	64.8	2.78	0.43
*Taxodium distichum*	6	16.59 (3.25) 12.45–21.59	2.07 (0.76) 0.73–2.99	0.35 (0.02) 0.32–0.38	0.4 (0.14) 0.23–0.57	6.5	2.13	0.42
*Thuja occidentalis*	13	25.2 (14.6) 5.84–50.29	15.86 (18.5) 0.58–56.09	0.28 (0.03) 0.24–0.34	0.74 (0.12) 0.53–0.95	55	3.45	0.29
*Tsuga canadensis*	82	25.68 (18.25) 3.81–82.04	17.23 (21.65) 0.29–145.24	0.41 (0.03) 0.31–0.5	0.74 (0.1) 0.41–0.91	60	4.83	0.38
*Tsuga heterophylla*	22	43.53 (15.69) 21.84–69.85	51.23 (44.63) 2.01–142.64	0.45 (0.04) 0.35–0.52	0.72 (0.19) 0.2–0.93	66	4.96	0.42
Hardwoods (species = 36; *N* = 1677)								
*Acer nigrum*	4	35.31 (28.22) 4.06–67.06	19.69 (21.55) 0.54–45.22	0.61 (0.08) 0.55–0.72	0.65 (0.15) 0.47–0.84	5.5	3	0.52
*Acer rubrum*	196	19.3 (11.33) 3.05–85.6	5.63 (8.63) 0.06–72.11	0.5 (0.05) 0.39–0.65	0.54 (0.15) 0.14–0.91	5.6	3.44	0.49
*Acer saccharinum*	14	28.47 (10.87) 13.72–49.02	8.5 (6.62) 1.91–22.04	0.49 (0.03) 0.44–0.54	0.55 (0.11) 0.38–0.77	5.5	3.6	0.44
*Acer saccharum*	87	21.86 (17.02) 5.08–83.31	8.28 (13.89) 0.06–79.63	0.61 (0.04) 0.48–0.68	0.61 (0.13) 0.32–0.9	5.5	4.76	0.56
*Betula alleghaniensis*	2	13.84 (0.9) 13.21–14.48	2.36 (2.25) 0.77–3.95	0.54 (0.04) 0.51–0.57	0.77 (0.01) 0.76–0.78	5.5	3.17	0.55
*Betula papyrifera*	3	18.8 (3.91) 15.49–23.11	2.45 (1.46) 0.77–3.37	0.53 (0.02) 0.51–0.55	0.61 (0.17) 0.48–0.81	5	1.54	0.48
*Carpinus caroliniana*	18	10.37 (4.04) 4.06–16	2.38 (1.86) 0.45–6.35	0.56 (0.02) 0.53–0.6	0.56 (0.15) 0.32–0.81	5.5	4.58	0.58
*Carya cordiformis*	6	43.35 (18.59) 20.32–75.44	25.72 (12.19) 8.59–45.69	0.62 (0.02) 0.58–0.64	0.73 (0.11) 0.57–0.9	5.5	2.07	0.6
*Carya glabra*	5	10.26 (4.47) 6.35–16	1.39 (1.32) 0.52–3.67	0.62 (0.05) 0.57–0.66	0.71 (0.15) 0.5–0.86	5.5	2.69	0.66
*Celtis occidentalis*	10	28.37 (16.87) 5.59–54.36	9.52 (6.5) 0.25–18.9	0.55 (0.03) 0.5–0.59	0.64 (0.16) 0.39–0.94	5.5	3.17	0.49
*Cercis canadensis*	2	4.7 (0.9) 4.06–5.33	0.68 (0.19) 0.54–0.82	0.57 (0.02) 0.56–0.59	0.6 (0.2) 0.46–0.75	6	3	0.65
*Cornus florida*	6	8.42 (2.39) 4.32–11.18	1.63 (0.8) 0.45–2.9	0.64 (0.03) 0.59–0.67	0.49 (0.16) 0.3–0.73	5.6	4.87	0.64
*Fagus grandifolia*	21	36.44 (24.13) 5.08–79.76	18.39 (17.73) 0.42–63.04	0.6 (0.04) 0.52–0.71	0.73 (0.14) 0.48–0.93	6	4.75	0.56
*Fraxinus americana*	6	39.88 (26.49) 11.18–81.79	11.48 (12.77) 0.99–32.6	0.55 (0.05) 0.5–0.6	0.5 (0.12) 0.35–0.64	5.7	2.46	0.55
*Ilex opaca*	1	18.8 (NA) 18.8–18.8	3.81 (NA) 3.81–3.81	0.47 (NA) 0.47–0.47	0.48 (NA) 0.48–0.48	42	4.28	0.5
*Juglans nigra*	1	28.96 (NA) 28.96–28.96	5.26 (NA) 5.26–5.26	0.52 (NA) 0.52–0.52	0.63 (NA) 0.63–0.63	5	1.93	0.51
*Liquidambar styraciflua*	326	22.07 (10.98) 4.32–50.8	4.66 (4.77) 0.45–34.25	0.48 (0.03) 0.28–0.56	0.45 (0.12) 0.19–0.92	6	1.59	0.46
*Liriodendron tulipifera*	110	28.68 (15.9) 4.57–80.52	7.82 (7.09) 0.45–33.24	0.43 (0.04) 0.31–0.51	0.52 (0.13) 0.2–0.89	5.3	2.07	0.35
*Magnolia virginiana*	5	22.71 (6.69) 16.76–32.51	4.54 (4.34) 1.41–11.97	0.41 (0.04) 0.36–0.44	0.39 (0.08) 0.3–0.48	12	3	0.42
*Nyssa sylvatica*	21	26.6 (10.89) 6.6–45.21	8.23 (6.29) 0.37–24.36	0.53 (0.04) 0.43–0.6	0.56 (0.13) 0.35–0.83	5.5	3.52	0.46
*Oxydendrum arboreum*	8	12.19 (6.05) 4.06–22.35	1.43 (1.07) 0.45–3.49	0.52 (0.03) 0.47–0.55	0.55 (0.09) 0.4–0.73	6	2.7	0.5
*Platanus occidentalis*	28	31.94 (11.18) 6.1–49.78	15.89 (11.73) 0.86–43.41	0.46 (0.03) 0.39–0.51	0.47 (0.1) 0.33–0.74	6	2.86	0.42
*Populus tremuloides*	80	23.96 (11.43) 5.33–60.45	4.03 (6.43) 0.09–34.28	0.4 (0.05) 0.25–0.54	0.51 (0.16) 0.22–0.86	4.9	1.21	0.35
*Prunus serotina*	20	12.92 (8.61) 4.32–35.56	2.31 (2.55) 0.45–10.28	0.56 (0.04) 0.5–0.63	0.5 (0.16) 0.23–0.8	5.5	2.46	0.47
*Quercus alba*	234	24.47 (15.23) 4.32–87.63	14.07 (18.25) 0.45–91.04	0.64 (0.05) 0.43–0.76	0.56 (0.13) 0.28–0.95	5.5	2.85	0.63
*Quercus coccinea*	76	28.77 (12.53) 4.57–56.39	18.86 (18.18) 0.91–82.33	0.59 (0.04) 0.51–0.69	0.55 (0.11) 0.19–0.88	5.7	2.07	0.62
*Quercus falcata*	42	29.05 (10.95) 7.62–47.75	17.04 (14.19) 1.04–57.56	0.59 (0.03) 0.51–0.65	0.47 (0.12) 0.24–0.69	8.2	2.5	0.58
*Quercus laurifolia*	16	20.32 (8.05) 4.06–35.31	6.57 (5.71) 0.54–17.83	0.6 (0.04) 0.55–0.71	0.49 (0.14) 0.31–0.84	8.4	3.34	0.58
*Quercus nigra*	119	22.9 (11.47) 3.81–50.8	12.79 (13.58) 0.5–62.55	0.59 (0.03) 0.47–0.66	0.5 (0.12) 0.22–0.96	8.6	2.24	0.63
*Quercus prinus*	59	27.88 (15.79) 8.38–67.31	13.62 (15.86) 0.86–66.73	0.62 (0.04) 0.52–0.69	0.53 (0.13) 0.23–0.87	8.2	2.85	0.57
*Quercus rubra*	44	31.18 (21.47) 4.32–83.31	12.85 (15.94) 0.24–67.3	0.58 (0.05) 0.46–0.7	0.54 (0.17) 0.23–0.9	5.5	2.75	0.56
*Quercus stellata*	28	25.01 (11.7) 7.62–53.09	10.17 (9.96) 0.82–36.2	0.66 (0.04) 0.57–0.72	0.57 (0.11) 0.39–0.79	7.9	2.16	0.66
*Quercus velutina*	43	27.21 (14.78) 3.3–55.37	11.07 (11.42) 0.45–44.59	0.59 (0.05) 0.51–0.69	0.51 (0.12) 0.24–0.84	5.5	2.72	0.56
*Robinia pseudoacacia*	18	27.81 (8.64) 14.22–41.66	8.17 (5.51) 1.81–20.09	0.65 (0.03) 0.6–0.69	0.48 (0.15) 0.25–0.75	4.6	1.72	0.67
*Sassafras albidum*	1	7.87 (NA) 7.87–7.87	0.45 (NA) 0.45–0.45	0.52 (NA) 0.52–0.52	0.26 (NA) 0.26–0.26	5.5	1.68	0.42
*Tilia americana*	17	37.04 (19.57) 6.6–71.37	11.89 (11.07) 0.41–43.41	0.35 (0.03) 0.27–0.4	0.6 (0.12) 0.38–0.88	5.5	3.98	0.32

### Tree sample data selection

We selected trees from the combined database having measured leaf mass (M_l_); stem DBH; wood specific gravity (SG), which is the “basic” density (g/cm^3^) of the wood on an oven‐dried weight and a green volume basis divided by the density of water; live crown ratio (LCR), which is the length of live crown divided by the total height of the tree, and crown class (CC). CC was assigned to each tree based on the position of the tree in the canopy—(1) overtopped, (2) intermediate, (3) codominant, (4) dominant, or (5) open grown—and was used to indicate shading experienced by the tree (MacFarlane & Kane, 2017). Trees that did not contain at least two significant figures in each of the numeric variables were removed from the study. This resulted in a data set of 3628 trees of 61 species (36 hardwoods and 25 softwoods) (Table 1). Additionally, each tree was assigned a published value of specific gravity (SG_p_) from Chave et al. (2009), with the Dryad data package from Zanne et al. (2014). The latter was to determine whether a published value could be substituted for measured values of SG calculated from samples taken at breast height (as suggested by MacFarlane, 2015), which would be impractical to obtain for every tree in the US NFI.

Trees were assigned species‐specific leaf longevity (LL) values based on published averages from the GLOPNET database (Wright et al., 2004), available from the TRY plant database (Kattge et al., 2011), as well as published values from Hallik et al. (2009), Niinemets and Lukjanova (2003), Pinchot (1907), Balster and Marshall (2000), Gower et al. (1989), and Reich et al. (1999). Trees were assigned species‐specific shade tolerance (ST) ratings on a scale of 1 (shade intolerant) to 5 (shade tolerant) using published values from Niinemets and Valladares (2006).

The geographic coordinates of the sample locations were used in conjunction with 2.5‐min (~50 km^2^) resolution WorldClim global average monthly climate data from 1970 to 2000 (Fick & Hijmans, 2017) to assign mean annual temperature (MAT) (°C) and mean annual precipitation (MAP) (mm) to each tree. These variables were used to represent biological (DBH, LCR, CC), species‐specific (SG, LL, ST), and environmental (MAP, MAT) factors determining foliage mass.

### Model formulation and variable selection

The initial model followed a form similar to that recommended by Dettmann and MacFarlane (2019). However, due to insufficient data on tree growth rates and neighboring trees for many of the trees in this study, the variables of basal area increment and competition index from the Dettmann and MacFarlane (2019) model were not included in the model. Instead, MAP (mm) and MAT (°C) were added to account for gross variation in climate across sites, resulting in the following model:

(1) $$\begin{array}{c} \ln\left(M_{l}\right)=\beta_{0}+\beta_{1}\ln\left(\mathrm{DBH}\right)+\beta_{2}\ln\left(\mathrm{LL}\right)+\beta_{3}\ln\left(\mathrm{LCR}\right)+\beta_{4}\ln\left(\mathrm{SG}\right)+\\ \beta_{5}\ln\left(\mathrm{ST}\right)+\beta_{6}\ln\left(\mathrm{MAT}+30\right)+\beta_{7}\ln\left(\mathrm{MAP}\right)+Ɛ \end{array}$$

β_0_ is a variable intercept based on CC that takes different values for the canopy positions of open grown, dominant, codominant, intermediate, and overtopped. β_1−_β_7_ are coefficients for variables of DBH, LL, LCR, SG, ST, MAT, and MAP, respectively, and Ɛ represents the error term.

Variables in Equation (1) were log transformed because the relationships between leaf mass and predictors were generally observed to be heteroscedastic, and this allowed for nonlinear trends to be captured, but with the benefits of a linear modeling framework (Appendix S1: Figure S1). To achieve the log transformation with MAT whose values could be negative, MAT was translated by adding 30 to each value; 30 was selected for the translation so as to ensure that a positive result was obtained for even the minimum MAT value.

An alternative to Equation (1) included published SG (SG_p_), rather than measured SG, to address the concern that it would be very unlikely that SG would be measured on every tree as part of any NFI:

(2) $$\begin{array}{c} \ln\left(M_{l}\right)=\beta_{0}+\beta_{1}\ln\left(\mathrm{DBH}\right)+\beta_{2}\ln\left(\mathrm{LL}\right)+\beta_{3}\ln\left(\mathrm{LCR}\right)+\beta_{4}\ln\left({\mathrm{SG}}_{p}\right)+\\ \beta_{5}\ln\left(\mathrm{ST}\right)+\beta_{6}\ln\left(\mathrm{MAT}+30\right)+\beta_{7}\ln\left(\mathrm{MAP}\right)+Ɛ \end{array}$$

Equations (1) and (2) were fit to the data set using backwards stepwise regression using the package MASS (Venables & Ripley, 2002) in the R (R Core Team, 2019) environment to find the optimum model to estimate the dry mass of leaves.

Though we were seeking the optimum model, we also retained simpler models—reduced versions of Equation (1) or (2)—as potential candidates, because we wanted to see how much predictive power would be lost by dropping certain variables, especially those that would be more costly to measure or acquire, since an important part of this research was to create a model that could be broadly implemented for the US NFI. Four more models were selected to represent models of varying complexity and differing data collection limitations:

(3) $$\ln\left(M_{l}\right)=\beta_{0}+\beta_{1}\ln\left(\mathrm{DBH}\right)+Ɛ$$

(4) $$\ln\left(M_{l}\right)=\beta_{0}+\beta_{1}\ln\left(\mathrm{DBH}\right)+\beta_{3}\ln\left(\mathrm{LCR}\right)+Ɛ$$

(5) $$\ln\left(M_{l}\right)=\beta_{0}+\beta_{1}\ln\left(\mathrm{DBH}\right)+\beta_{3}\ln\left(\mathrm{LCR}\right)+\beta_{6}\ln\left(\mathrm{MAT}+30\right)+\beta_{7}\ln\left(\mathrm{MAP}\right)+Ɛ$$

(6) $$\ln\left(M_{l}\right)=\beta_{0}+\beta_{1}\ln\left(\mathrm{DBH}\right)+\beta_{2}\ln\left(\mathrm{LL}\right)+\beta_{3}\ln\left(\mathrm{LCR}\right)+\beta_{5}\ln\left(\mathrm{ST}\right)+\beta_{6}\ln\left(\mathrm{MAT}+30\right)+\beta_{7}\ln\left(\mathrm{MAP}\right)+Ɛ$$

Collinearity of the variables included in the models was evaluated using a variable inflation factor (VIF). No VIF in the fully specified model Equation (2) was over 5, so no major concerns of multicollinearity between our predictor variables was determined (Appendix S1: Table S1).

### Model comparisons, validation, and assessment

Four types of error metric approaches were used to compare models to each other, with mean percentage error (MPE), mean absolute error (MAE), mean absolute percentage error (MAPE), and mean arctangent absolute percentage error (MAAPE) calculated for all models fitted as follows:

$$\mathrm{MPE}=\frac{1}{n}\times \sum_{t=1}^{n}\frac{p_{t}-a_{t}}{a_{t}}\times 100$$

$$\mathrm{MAE}=\frac{1}{n}\times \sum_{t=1}^{n}\left|p_{t}-a_{t}\right|$$

$$\text{MAPE}=\frac{1}{n}\times \sum_{t=1}^{n}\frac{\left|p_{t}-a_{t}\right|}{a_{t}}\times 100$$

$$\text{MAAPE}=\frac{1}{n}\times \sum_{t=1}^{n}\arctan\left(\frac{\left|p_{t}-a_{t}\right|}{a_{t}}\right)\times 100$$

where *n* is the total number of trees, *a*_*t*_ the observed foliage mass, and *p*_*t*_ the estimated foliage mass from the model for tree *t*. These error metrics were calculated using the nontransformed observed values and on back‐transformed estimated values using the correction factor given by Baskerville (1972).

The first validation method used was a 10‐fold cross validation. The second method of validation was done using a k‐fold cross validation where one species was removed from data at a time and the model was refitted; we called this “leave‐one‐species‐out” cross validation. The leave‐one‐species‐out cross validation was done to compare how each model might perform on species where no data were observed. Models were then compared to one another using MPE, MAE, MAPE, MAAPE, their adjusted *R* ^2^, and Akaike's information criterion (AIC).

To further understand the relative contributions of predictors to model accuracy, we calculated the relative importance of each variable using dominance analysis (Budescu, 1993; Johnson & Lebreton, 2004). This gave the proportion of the model‐explained variance into nonnegative contributions from each variable based on sequential *R* ^2^ values, but it accounts for the dependence of the order in which the variables are added by averaging over orderings (Grömping, 2006).

All model development and statistical analyses were carried out using R version 3.6.1 (R Core Team, 2019), with the packages MASS (Ripley et al., 2019) and lme4 (Bates et al., 2019) in model generation; raster (Hijmans et al., 2019), sp (Pebesma et al., 2019), XML (CRAN Team et al., 2019), and relaimpo (Groemping & Matthias, 2018) in calculating relative importance; caret (Kuhn et al., 2019) in data management and processing; and stargazer (Hlavac, 2018), ggplot2 (Wickham et al., 2019), ggsn (Baquero, 2019), maps (Becker et al., 2018), cowplot (Wilke, 2019), and gridExtra (Auguie & Antonov, 2017) in data display and plotting.

## RESULTS

### Variables influencing leaf mass

Interaction terms were initially considered, and multiple interactions were found to be significant (at the 0.05 level). Despite this, these terms added very little in explanatory power (adjusted *R* ^2^ increase of 0.02). Owing to the lack of value added in terms of model fit and parsimony, interaction terms were removed from further consideration.

**FIGURE 2:**
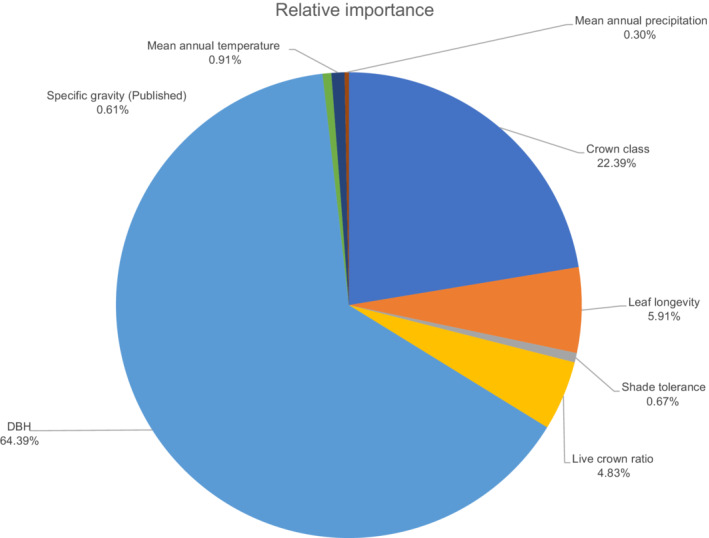
Relative importance of variables for prediction of leaf mass rounded to nearest 0.01% of ordinary least squares model with diameter at breast height (DBH), specific gravity as given by Chave et al. (2009) (SG_p_), mean annual temperature (MAT), mean annual precipitation (MAP), crown classification (CC), leaf longevity (LL), shade tolerance (ST), and uncompacted live crown ratio (LCR). The relative importance gives the proportion of model explained variance into nonnegative contributions from each variable based on sequential *R* ^2^ but accounts for the dependence on the order the variables were added by averaging over all possible orderings (Grömping, 2006).

The coefficients for each of the models explored are shown in Table 3. All initial variables from both Equations (1) and (2) were retained when subjected to backward stepwise regression, using AIC as the criterion, indicating all model variables contributed significantly to leaf biomass. Figure 2 shows the relative importance of each variable in the model containing DBH, LCR, ST, LL, CC, SG_p_, MAT, and MAP in Equation (2). The most important predictor was tree size (DBH), with a relative importance of 64.39% overall, followed by a tree's CC at 22.39%. The next most important variables were LL (5.91%) and LCR (4.83%). The remaining variables of SG_p_, ST, MAT, and MAP each held much lower relative importance, with values of 0.61%, 0.67%, 0.91%, and 0.30%, respectively Figure (2).

**TABLE 3:** Model coefficient and associated standard error in parentheses. Models were fitted using all 3901 tree samples. Each model contains selected variations of complexity with model Equations (1) and (2) being the result of backward stepwise regression on all variables, with field‐measured or published values for specific gravity, respectively. Crown class serves as a variable intercept for all models but Equation (3).

	Dependent variable: log dry leaf mass (kg)					
Independent variable	Equation (1)	Equation (2)	Equation (3)	Equation (4)	Equation (5)	Equation (6)
Log diameter at breast height (DBH) (cm)	1.703^***^ (0.017)	1.708^***^ (0.017)	1.825^***^ (0.018)	1.647^***^ (0.021)	1.679^***^ (0.021)	1.692^***^ (0.017)
Log crown ratio	0.755^***^ (0.028)	0.750^***^ (0.028)	…	0.593^***^ (0.030)	0.775^***^ (0.033)	0.748^***^ (0.028)
Log leaf longevity (month)	0.413^***^ (0.010)	0.406^***^ (0.010)	…	…	…	0.346^***^ (0.009)
Log shade tolerance	0.236^***^ (0.024)	0.221^***^ (0.024)	…	…	…	0.254^***^ (0.024)
Log specific gravity (study observed values)	0.689^***^ (0.053)	…	…	…	…	…
Log specific gravity (published values)	…	0.800^***^ (0.054)	…	…	…	…
Log mean annual temperature (°C + 30)	2.049^***^ (0.114)	1.701^***^ (0.118)	…	…	1.063^***^ (0.129)	2.198^***^ (0.116)
Log mean annual precipitation (mm)	−0.191^***^ (0.038)	−0.180^***^ (0.037)	…	…	−0.009 (0.044)	−0.156^***^ (0.039)
Intercepts						
Intercept	…	…	−3.711^***^ (0.054)	…	…	…
Crown class						
Open grown	−9.947^***^ (0.334)	−8.632^***^ (0.360)	…	−2.490^***^ (0.066)	−6.442^***^ (0.365)	−11.100^***^ (0.329)
Dominant	−9.789^***^ (0.044)	−8.456^***^ (0.043)	…	−2.567 (0.053)	−6.482 (0.052)	−9.110^***^ (0.045)
Codominant	−9.932 (0.041)	−8.601 (0.041)	…	−2.678^***^ (0.049)	−6.584^***^ (0.049)	−11.046 (0.042)
Intermediate	−10.176^***^ (0.042)	−8.839^***^ (0.042)	…	−3.004^***^ (0.049)	−6.874^***^ (0.049)	−11.301^***^ (0.043)
Suppressed	−10.346^***^ (0.042)	−9.009^***^ (0.042)	…	−3.089^***^ (0.050)	−6.970^***^ (0.050)	−11.473^***^ (0.043)

*** *p* < 0.01.

As expected, larger trees (DBH) with longer crowns (LCR) had more foliage mass. Specific gravity also had a positive relationship with total leaf mass. Both of the life history traits included (ST and LL) showed a positive relationship with foliage mass, indicating that trees with longer lived leaves and increased tolerance to shade maintained greater leaf masses, holding other factors constant. When included, MAT also had a significant and positive relationship with leaf mass. MAP, on the other hand, was negatively related to leaf biomass, when all other factors were included. Thus, holding other factors constant, trees in warmer, drier climates maintain a greater leaf mass.

Table 4 shows fit statistics for all the models explored. Equations (1) and (2) were the best models and very similar statistically, though the model fitted with SG_p_ performed slightly better with regard to AIC (partial dependency plots for Equation 2 can be found in Appendix S1: Figure S2). The remaining models showed increasingly poorer performance in this order: Equation (6) > Equation (5) > Equation (4) > Equation (3). For all models (Table 4), the leave‐one‐species‐out cross validation showed relatively small differences between MPE, MAE, MAPE, and MAAPE from that derived from the 10‐fold cross‐validation approach, indicating a general tendency of the models to work as well for species not included during model development (Table 5).

**TABLE 4:** Fit statistics (for log‐transformed model and data) and cross‐validation results of mean percentage error (MPE) (%), mean absolute error (MAE) (kg), and mean absolute percentage error (MAPE) (%) for model Equations (1)–(6). Models were fitted using all 3628 tree samples. Each model contains selected variations of complexity with model Equations (1) and (2) being the result of stepwise regression on all variables and specific gravity reported by studies where data were taken and published values of specific gravity, respectively.

Model	Adjusted *R* ^2^	Root mean square error	Akaike's information criterion	10‐fold cross validation				Leave‐one‐species‐out cross validation			
				MPE	MAE	MAPE	MAAPE	MPE	MAE	MAPE	MAAPE
Equation (1)	0.86	0.50	5338.36	29.98	4.76	51.58	0.39	31.63	5.05	53.83	0.4
Equation (2)	0.87	0.50	5287.48	29.67	4.6	51.05	0.38	31.82	4.84	53.41	0.39
Equation (3)	0.74	0.69	7670.81	67.15	5.92	91.38	0.52	68.48	6.04	93.24	0.52
Equation (4)	0.79	0.63	6925.52	52.51	5.59	76.17	0.47	52.34	5.82	79.02	0.48
Equation (5)	0.80	0.62	6809.51	49.26	5.56	72.81	0.46	51.49	5.95	78.74	0.48
Equation (6)	0.86	0.51	5500.63	31.75	4.79	53.49	0.39	33.38	5.08	56.09	0.41

**TABLE 5:** Cross‐validation results from leave‐one‐species‐out cross validation of mean error, mean percentage error (MPE), and mean absolute percentage error (MAPE) for model Equation (2) for each species.

Species	Species code	*N*	Mean error	MPE	MAPE
Softwoods					
*Abies balsamea*	12	50	2.55	42.51	62.36
*Abies concolor*	15	36	0.59	24.70	54.35
*Abies grandis*	17	20	−25.32	−31.97	31.97
*Abies lasiocarpa*	19	28	−5.60	−14.05	33.77
*Juniperus virginiana*	68	2	−3.15	−41.70	41.70
*Larix laricina*	71	6	−4.37	−45.65	45.65
*Larix occidentalis*	73	24	2.31	44.85	54.61
*Picea engelmannii*	93	51	−8.60	−9.22	38.93
*Picea glauca*	94	2	−1.58	−7.49	13.16
*Pinus contorta*	108	61	1.48	31.18	54.73
*Pinus echinata*	110	63	0.92	61.95	62.15
*Pinus elliottii*	111	540	−0.33	31.33	45.56
*Pinus glabra*	115	1	0.99	34.61	34.61
*Pinus palustris*	121	151	1.68	37.31	47.95
*Pinus ponderosa*	122	135	−6.95	−12.01	40.37
*Pinus resinosa*	125	13	5.02	37.00	38.17
*Pinus rigida*	126	31	0.18	81.01	91.20
*Pinus strobus*	129	137	3.71	42.06	58.16
*Pinus taeda*	131	242	−1.82	0.91	33.95
*Pinus virginiana*	132	84	0.71	22.25	42.54
*Pseudotsuga menziesii*	202	152	15.94	54.40	63.46
*Taxodium distichum*	221	12	2.41	211.99	212.89
*Thuja occidentalis*	241	13	0.17	9.64	31.08
*Tsuga canadensis*	261	82	10.46	77.10	84.86
*Tsuga heterophylla*	263	22	2.84	71.19	95.47
Hardwoods					
*Acer nigrum*	314	4	−0.67	0.91	39.51
*Acer rubrum*	316	201	0.55	43.34	64.23
*Acer saccharinum*	317	14	2.55	35.89	46.13
*Acer saccharum*	318	87	1.41	64.32	80.53
*Betula alleghaniensis*	371	2	0.06	68.48	97.85
*Betula papyrifera*	375	3	0.05	85.79	133.88
*Carpinus caroliniana*	391	18	−0.48	−6.80	34.99
*Carya cordiformis*	402	6	4.06	5.09	44.88
*Carya glabra*	403	5	0.51	90.80	100.81
*Celtis occidentalis*	462	10	3.38	16.64	50.54
*Cercis canadensis*	471	5	−0.10	−14.40	14.40
*Cornus florida*	491	8	−0.35	−20.83	33.33
*Fagus grandifolia*	531	25	10.24	31.10	48.91
*Fraxinus americana*	541	6	7.51	65.13	69.15
*Ilex opaca*	591	1	6.51	170.76	170.76
*Juglans nigra*	602	1	5.16	98.05	98.05
*Liquidambar styraciflua*	611	452	2.03	60.21	69.01
*Liriodendron tulipifera*	621	129	0.75	7.35	30.83
*Magnolia virginiana*	653	6	2.63	126.30	126.30
*Nyssa sylvatica*	693	21	−1.35	4.77	30.90
*Oxydendrum arboreum*	711	8	0.86	65.06	84.67
*Platanus occidentalis*	731	29	−4.11	14.12	62.32
*Populus tremuloides*	746	80	−0.18	73.73	104.08
*Prunus serotina*	762	26	0.28	3.21	34.77
*Quercus alba*	802	238	−1.46	15.25	42.68
*Quercus coccinea*	806	76	−6.04	−9.31	43.27
*Quercus falcata*	812	51	−1.75	7.10	33.40
*Quercus laurifolia*	820	39	1.86	67.55	74.58
*Quercus nigra*	827	161	−0.61	18.84	45.21
*Quercus prinus*	832	71	1.28	28.66	41.07
*Quercus rubra*	833	49	3.23	29.42	56.93
*Quercus stellata*	835	28	4.01	56.81	59.37
*Quercus velutina*	837	44	−0.45	18.03	47.15
*Robinia pseudoacacia*	901	19	0.70	20.62	33.48
*Sassafras albidum*	931	3	−0.09	−20.08	20.08
*Tilia americana*	951	17	0.49	7.07	29.89

**FIGURE 3:**
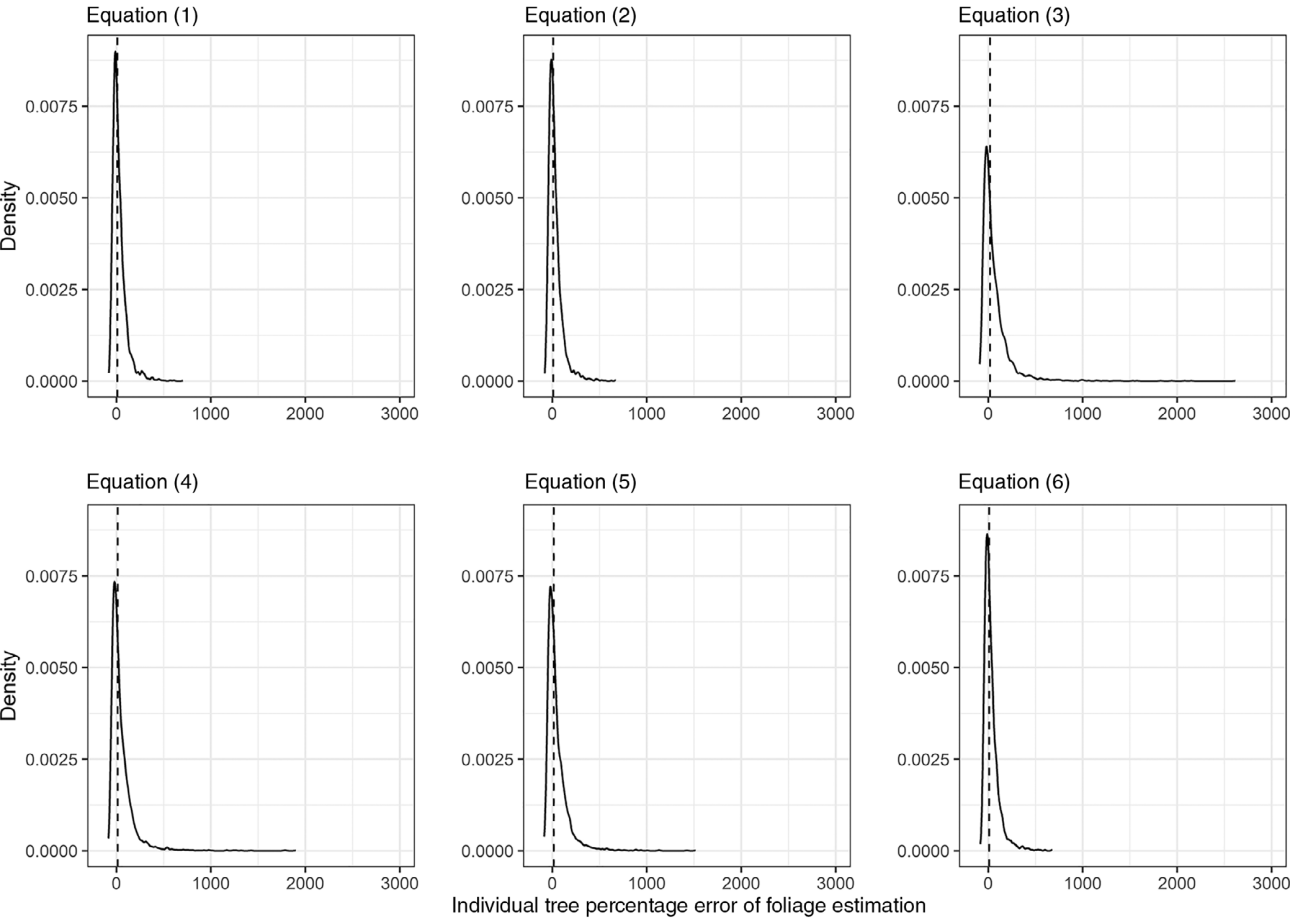
Probability density distribution of individual tree prediction error (%) of foliage estimation for Equations (1)–(6). The dashed vertical line is the median of the mean percentage error (MPE) distribution for each equation: Equation (1) = 9.82%, Equation (2) = 9.34%, Equation (3) = 19.83%, Equation (4) = 14.25%, Equation (5) = 15.07%, and Equation (6) = 10.01%. Positive values mean overestimation of measured leaf mass.

All the models showed a mean tendency to overestimate leaf mass ranging from ~30% to ~67% MPE across all models in the 10‐fold cross validation. However, the distribution of MPEs (Figure 3) showed that the models estimated leaf mass well for most of the population of trees (tall peaks near 0% MPE) (Figure 3), with very large overestimation errors for a relatively small subset of trees (right‐skewed distributions) (Figure 3), which heavily skewed the means. This skewness meant that the median prediction error was much lower than the mean (compare Figure 3 to Table 4), which was noticeably greater for Equations (3), (4), and (5) than for Equations (1), (2), and (6). In terms of absolute mass, the MAE from 10‐fold cross validation ranged from 4.60 to 5.92 kg compared to a mean leaf mass of 11.97 kg, indicating an approximate relative error range of around ±37%–50% of tree leaf mass, whereas the median MPE ranged between 9% and 19% (Figure 3).

## DISCUSSION

### Influence of tree size and crown shading on leaf mass estimation

Of the variables that we studied, DBH was the most important for predicting a tree's leaf mass. This was expected given well‐documented allometric scaling relationships between DBH and other components of tree mass, including foliage (Chojnacky et al., 2013; Clough et al., 2016; Wirth et al., 2004). The relative importance of DBH proved to be the largest of any predictor examined (64.39%) but yielded a percentage error and bias in prediction of foliage mass roughly double those of the best models tested (Table 4).

The next most important variable was CC (22.39%), suggesting that the position of a tree in the canopy in relation to others plays an important role in determining its leaf mass. Such a finding corroborates the findings from a study by Le Goff et al. (2004), where different measures of competition were correlated with leaf mass. Here, our categorical variable of CC appeared to encompass some of the effects of how trees are able to alter the resources available to neighbors (Palik et al., 1997). A tree's position in the canopy reflects resources available to it (Canham et al., 2004), and it' in addition to crown shape (MacFarlane et al., 2003; Muth & Bazzaz, 2003). Competition experienced can also alter expressed leaf traits such as leaf mass per area due to light availability (Ellsworth & Reich, 1993).

LCR was another important factor for estimating leaf mass, and other studies have confirmed this finding (Loomis et al., 1966; Temesgen et al., 2011). LCR influences the scaling relationship between live woody mass (i.e., sapwood) and leaf mass in trees (Mäkelä & Valentine, 2006) and can serve as a proxy for vigor. This may explain why it was relatively more important in this study, where tree vigor was not measured directly, than was the study by Dettmann and MacFarlane (2019), where tree vigor (most recent 5‐ and 10‐year basal area growth). Likewise, LCR may be a proxy for competition experienced by a tree because it integrates the effect of crowding on the reduction of leaf mass via the reduction of LCR (Antos et al., 2010).

A major problem in the use of LCR for the US NFI would be the fact that the FIA program records a “compacted” crown ratio (CLCR) on all FIA plots (US Forest Service, 2018) but only records the LCR on a subset. The difference between LCR and CLCR can be as much as 17% and would require a model to translate CLCR to LCR (Toney & Reeves, 2009). Attempts to do so have shown significant bias and poor performance for trees of different types (Randolph, 2010); however, equations to convert CLCR are available across all four FIA regions, which could provide nationwide coverage of LCR (Westfall et al., 2019). Little additional effort is required to record LCR along with CLCR, and, considering the subjective nature of CLCR, there have been calls to have LCR measured on all plots in the US NFI (Monleon et al., 2004; Pollard et al., 2006).

### Species‐specific variables and the idea of a trans‐species model

The generalized leaf mass model is based on the idea of a transferrable model, where the model could work for any tree of any species for which LL, ST, and SG can be defined (Dettmann & MacFarlane, 2019). The recommended approach requires trees to be assigned values for LL, ST, and SG_p_ based on published species values, so species is present in the model, at least implicitly. One of the values of this type of trans‐species approach is that it allows prediction for a new species, for which there were no data or insufficient data to use for model development.

Together, the combined relative importance of LL and ST was small (~6.5% combined) in comparison to some other variables included, demonstrating that the large intraspecific variation in leaf mass allocation is dependent on other factors, such as size or competitive environment (Table 4 and Figure 3). The relative lower importance of LL and ST may be in part due to their error in attribution to each individual tree. These published values are only species averages with their own uncertainty, since LL and ST may vary across populations and life stages. Including these two variables, however, did reduce MPE and MAPE by approximately 20% and 25%, respectively, in both 10‐fold cross validation and leave‐one‐species‐out cross validation (Table 4). Thus, inclusion of LL and ST in the trans‐species model provided important information for differentiating among trees similar in size and competitive environment. Further, adding these traits helped to reduce overestimation bias (Figure 3), which may have been caused by model coefficients being more heavily weighted toward some species than others owing to an unequal representation of some species in the data (Table 2).

LL held a relative importance of 5.91% and served to place all trees on a meaningful continuous scale, directly related to leaf function. Here, trees with a greater leaf longevity show a tendency to maintain a greater leaf mass, consistent with at least one other study (Reich et al., 1992), likely due to the strong positive relationship between LL and leaf mass per area (LMA) (Osnas et al., 2013). Additionally, leaves with a longer LL must maintain their leaves longer to achieve a positive carbon balance (Reich, 1987), so leaf mass naturally accumulates for species with longer‐lived leaves.

Shade tolerance showed a lower relative importance (0.67%) compared to LL, with more shade‐tolerant trees having significantly more leaf mass. Callaway et al. (2000) also found that shade‐tolerant trees held a greater leaf mass. One explanation for this is that shade‐tolerant trees are able to maintain additional leaves in lower light environments, such as on lower branches, deeper within their crowns (Valladares & Niinemets, 2008). This leads shade‐tolerant trees to have deeper crowns (Canham et al., 1994), with efficient leaf display in lower canopy positions (Abrams & Kubiske, 1990; Canham, 1988). With many more species included in this trans‐species leaf mass model than in the original model by Dettmann and MacFarlane (2019; 61 vs. 17), LCR became a relatively more important predictor than ST; this suggests a fairly high degree of variation in LCR at any given level of ST, making LCR a more reliable predictor of leaf mass across many species. Similarly, the higher relative importance of CC than ST highlights how expression of shade tolerance is likely affected by an individual tree's light environment.

We found that published species‐specific values for SG were about as good a predictor as measured values, likely because they captured the mean trend of species‐specific, leaf mass–wood density relationships (Chave et al., 2009). Trees with higher SG (and SG_p_) tended to carry more foliage when accounting for other variables, though SG_p_ had a relative importance of only about 0.61%. Forrester et al. (2017) also found a positive relationship between foliage mass and wood density but noted a high degree of variability within and between species. This variation of wood specific gravity within (Lei et al., 1996) and between species (Chave et al., 2006) can be seen with measured values of SG (Table 2), while SG_p_ only represents the mean variation between species. Trees with denser wood have been noted to tolerate competition to a greater degree (Kunstler et al., 2015), so SG may be confounded with other variables in the model, such as CC.

### Climatic influences

Our new model captures general trends of how climate affects tree leaf mass accumulation, where leaf mass increases with increasing MAT and decreases with increasing MAP. Climatic variation was shown in other studies to correlate with leaf traits worldwide, with LMA being positively correlated with MAT and increasing as rainfall decreases (Wright et al., 2004). Here, both climatic variables showed a relatively low importance as predictors (0.91% and 0.30%, for MAT and MAP, respectively) compared to other variables. Thus, our results suggest that, for trees at different locations, experiencing different climatic conditions, the size and local competitive environment (expressed in CC) experienced by a tree are the dominant forces determining its leaf mass. Nonetheless, that should not be interpreted to indicate that climate does not have an important influence on tree leaf mass. Since trees are adapted to their local environments and exhibit plastic growth, it is likely that the other variables and functional traits related to life history already incorporate climate effect. Including climate variables was a significant improvement over measuring only the metrics of tree size DBH and LCR, so climate variables likely capture some general information about species, since even common species tend to specialize in certain climatic regions.

### Explanations for overestimation bias

The models showed a skewness toward overestimation bias, which bears greater discussion (Figure 3). We expect that this trend reflects both the variables selected and the underlying data. In terms of the variables selected, we can see that models which rely heavily on the size of the tree (here DBH and LCR) tended to produce the greatest overestimation bias (Equations 3 and 4, Figure 3). We assume that most of the trees sampled were relatively healthy when destructively sampled for foliage mass estimation, having a “full” complement of leaves for their size, but that some of the trees sampled were likely less vigorous, which would cause the model to be more likely to overestimate leaf mass for a tree based only on its size. We can see that the model with DBH alone had the largest skewness and adding LCR and CC, which might index competitive stress, reduced it somewhat (Figure 3).

Since we drew data from a large number of historical studies, we could not confirm that every study only selected “healthy” trees for its sampling. Further, our author group's extensive experience with tree biomass estimation from destructive sampling indicates that one is likely to underestimate biomass components because measurement errors are most likely to be omissions of biomass. For example, when a tree is felled, sampled too early in the growing season before leaves have time to fully initiate (Dettmann, 2017), or too late in the season when leaf mass may have been lost to senescence, leaves and needles may be lost. During examinations of the data, we removed some obvious underestimation outliers, but only from data the authors collected themselves; it was difficult to determine such outliers from historical data sets. Finally, alternative sampling methods have been used for determining foliage biomass, which can have a significant influence on the overall value (e.g., Temesgen et al., 2011). Nonetheless, skewness toward overestimation was reduced by adding more species‐specific functional traits to the model (cf. Equations 1, 2, and 6 to 3, 4, and 5 in Figure 3), suggesting the source of some of the estimation errors could be a result of relatively small sample sizes or unique leaf traits.

Some of the largest errors for species were found for *Taxodium distichum* (bald cypress), *Carya glabra* (pignut hickory), *Ilex opaca* (American holly), and *Magnolia virginiana* (sweetbay magnolia). Though we could not explain the exact sources of error for each species, *I. opaca* was the only broad‐leaved, evergreen species within our database, and there was only one sample from it. *T. distichum* was one of few deciduous coniferous species within our study, with only 12 samples. Additionally, MPE and MAPE, as *relative* error metrics, are often sensitive to even small prediction errors in small trees. Measures such as these can be inflated and skewed when many small trees are included in the sample. This pattern can be seen in the small sample specimens of *T. distichum*, which had mean errors >200% but leaf mass only averaging 2.4 kg. Thus, we may have lacked the necessary samples to fully describe species that lie outside of the norm of the most common deciduous broad‐leaved or evergreen, needle‐leaved tree species and overestimating for a subset of small trees, where published values of LL and ST are for mature trees. Another limitation revealed during our “leave‐one‐species‐out” cross validation was that there are many species with very few samples, where there is either only one individual or potentially only one site sampled. These were left in our analysis, however, because we were specifically interested in the transferability of the model and how it might perform for an undersampled or rare species.

Another potential source of error could be from the combination of so many data sources. Some variation could have crept in from variation in sampling methods, measurement error, or trees sampled when senescing or in decline. We conducted a simple cross validation on Model 2, leaving one study (author) out, which identified some studies that may be contributing more to the overall error (Appendix S1: Table S2). But disentangling each study from other confounding factors, such as species, geography, sampling method, and site variations, would be extremely difficult, if not impossible.

### Conclusion and recommendations for application

Overall, the trans‐species leaf mass model performed well for the vast majority of a wide variety of tree species across the large ecological domains of the United States. Our overall recommendation, given the results, would be to use Equation (2) as the best generalized foliage mass equation for the continental United States. Most of the tree‐level variables are already measured on all the NFI plots for the United States, with the exception of LCR (see *Discussion* section). The three species functional traits, ST, LL, and SG_p_, are available for a large number of species. Of 443 “forest‐growing” tree species listed in the FIA database (the database now includes many urban tree species), we were able to find ST values for 237 of them (about 53%) from Niinemets and Valladares (2006) and for all the tree species for this study, which included the most common or well‐studied species (most of the species in the Legacy tree data base).

Leaf longevity values specific to US tree species are relatively less abundant in the GLOPNET database (Wright et al., 2004), but we were able to find values for all the species in this study from additional sources (see *Methods*). This highlights the need to fill knowledge gaps related to ST and LL, which are unknown for many species found in large databases, such as the US NFI. In the case of LLs, it might be reasonable to assume similar values for congeneric species. It might also be possible to assign species reasonable ST values using qualitative descriptors found in textbooks on dendrology or silvicultural manuals, but it would be more valuable to expand the seminal methodology of Niinemets and Valladares (2006) to try to quantify ST for many more species.

Of the 443 forest tree species in the FIA database, all of them had published values for SG_p_. To use (measured) SG for biomass prediction on standing trees, cores would need to be either sampled from each individual tree or extracted from sample trees to obtain SG estimates for the population (Williamson & Wiemann, 2011). This could prove time consuming and expensive, and, given the small difference in predictive power between SG and SG_p_, it would appear that SG_p_ is sufficient. Similarly, Chave et al. (2005) recommended using SG_p_ for whole‐tree mass estimation models. However, MacFarlane (2015) found that measured SG could provide lower model error when used instead of SG_p_ in a formal assessment. In the latter case, the focus was on the woody components of total aboveground biomass, whereas this study is the first to test the difference between using SG_p_ and SG for predicting leaf mass.

Our overall conclusion is that the generalized model for leaf mass estimation we developed here captures the most important factors affecting leaf biomass variation between trees of the diverse tree species, with diverse life‐history traits, growing in a variety of climates in the United States and potentially in other parts of the world where information is available, such as Canada. As such, it appears that the model could be implemented to predict leaf biomass for approximately 227 of the 443 tree species listed in the FIA database, which have published values for ST and SG and for which approximate LL values can likely be obtained, if not already available. In the event that this is not sufficient, we also provided reduced versions of the trans‐species leaf mass model, which could also be used. There could also be a case for developing species‐specific models for species of direct interest for which there are large amounts of data and for which the data cover a significant portion of the species' spatial range. Yet for the case of species with little or no data on foliage mass, our model would be quite useful. In general, given the findings of this study, the trans‐species modeling approach deserves extension to other species, countries, and biomass components.

## CONFLICT OF INTEREST

The authors declare no potential conflict of interest related to this manuscript.

## Supporting information


Appendix S1

## Data Availability

Data (Radtke, 2016) are available from Virginia Tech's University Libraries at https://doi.org/10.7294/w4vd6wc6.
