# Supplementary material for: Testing a generalized leaf mass estimation method for diverse tree species and climates of the continental United States
Source: Ecol Appl. 2022 Jun 16;32(7):e2646. doi: 10.1002/eap.2646 (PMC9787613; doi:10.1002/eap.2646)

Appendix S1

Testing a generalized leaf mass estimation method for diverse tree species and climates of the continental United States

Garret T. Dettmann, David W. MacFarlane, Philip J. Radtke, Aaron R. Weiskittel, David L.R. Affleck, Krishna P. Poudel, and James Westfall

Ecological Applications

Table S1. The variable inflation factors for all variables included in equation 2.

Predictor Variable	Variable Inflation Factor
Log DBH (cm)	1.68
Log Crown Ratio	1.43
Log Leaf Longevity (mo)	1.33
Log Shade Tolerance	1.30
Log Specific Gravity (published values)	1.65
Log Mean Annual Temperature (°C + 30)	3.24
Log Mean Annual Precipitation (mm)	2.54
Crown Class	1.92

Table S2. Cross validation results from the leave one author out cross validation of mean error, mean percent error (MPE), and mean absolute percent error (MAPE) for model eq. 2 for each species.

Author	Mean Error	MPE	MAPE
Baldwin	-3.816	-15.305	25.865
Baldwin_and_Saucier	1.688	21.807	34.210
Clark	-0.892	19.723	47.321
Clark_Slsh_Complete_Tree	-2.548	-7.906	21.360
FIA_BIO_OSU	12.533	48.279	65.178
FIA_HF	4.243	65.534	79.303
FIA_ME	1.349	35.075	57.016
FIA_MI	6.788	31.417	52.592
FIA_MT	-5.345	21.707	62.054
Lohrey	-0.321	29.363	43.784
McNab_and_Clark	7.384	38.391	55.967
Mroz	1.474	117.953	125.082
NAU	-2.581	12.592	47.331
Phillips_and_McNab	0.656	59.112	70.316
VT	2.170	71.696	84.903
VT_CAL_CHECK	21.931	469.415	469.415
VT_OSU_OK	4.497	108.928	114.413
VT_POTLATCH	4.765	57.032	73.588

Figure S1. Residual plots for each proposed equation before and after applying the log-log transformation.

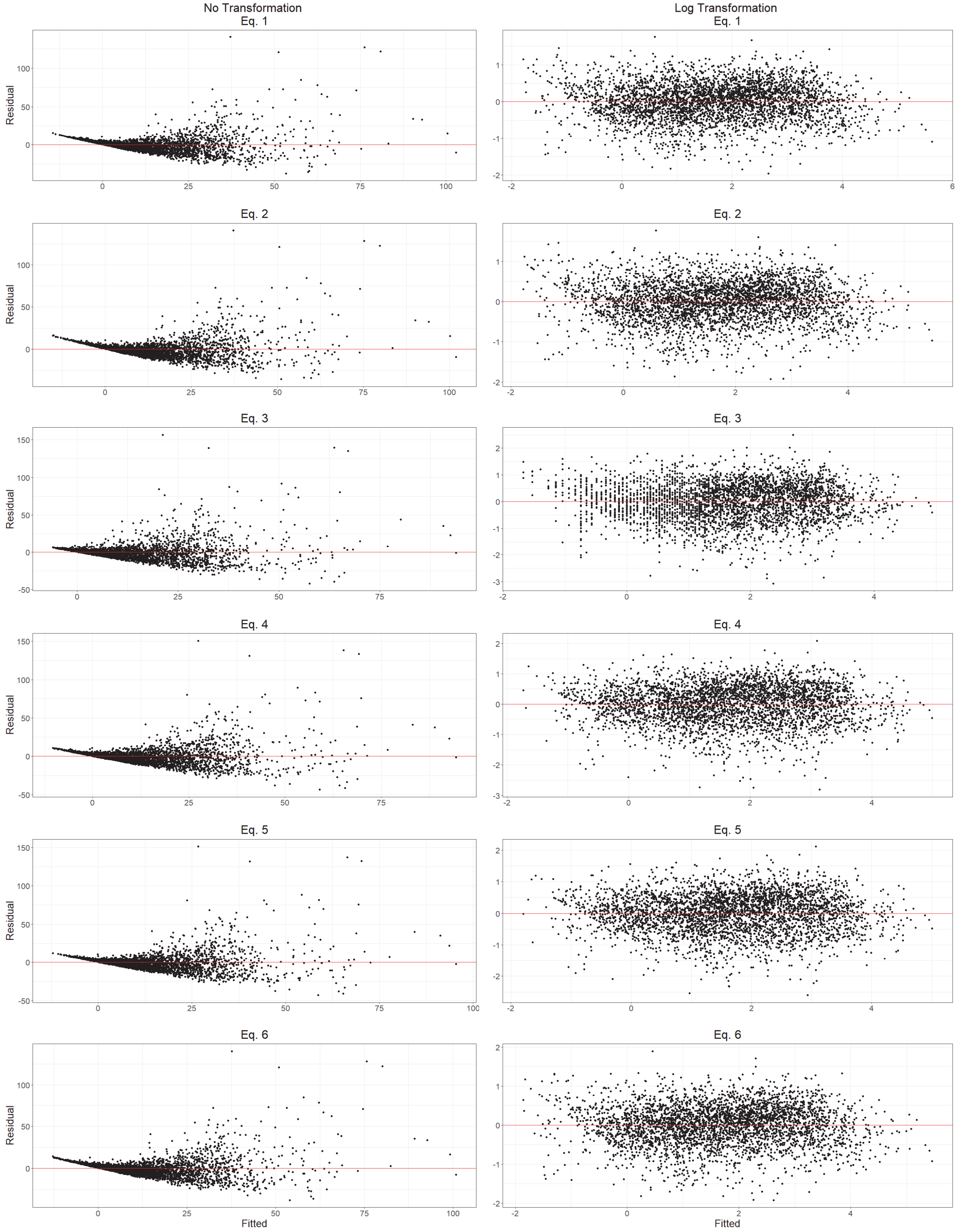

Figure S2. Partial dependency plots for equation 2 transformed back into original scale.

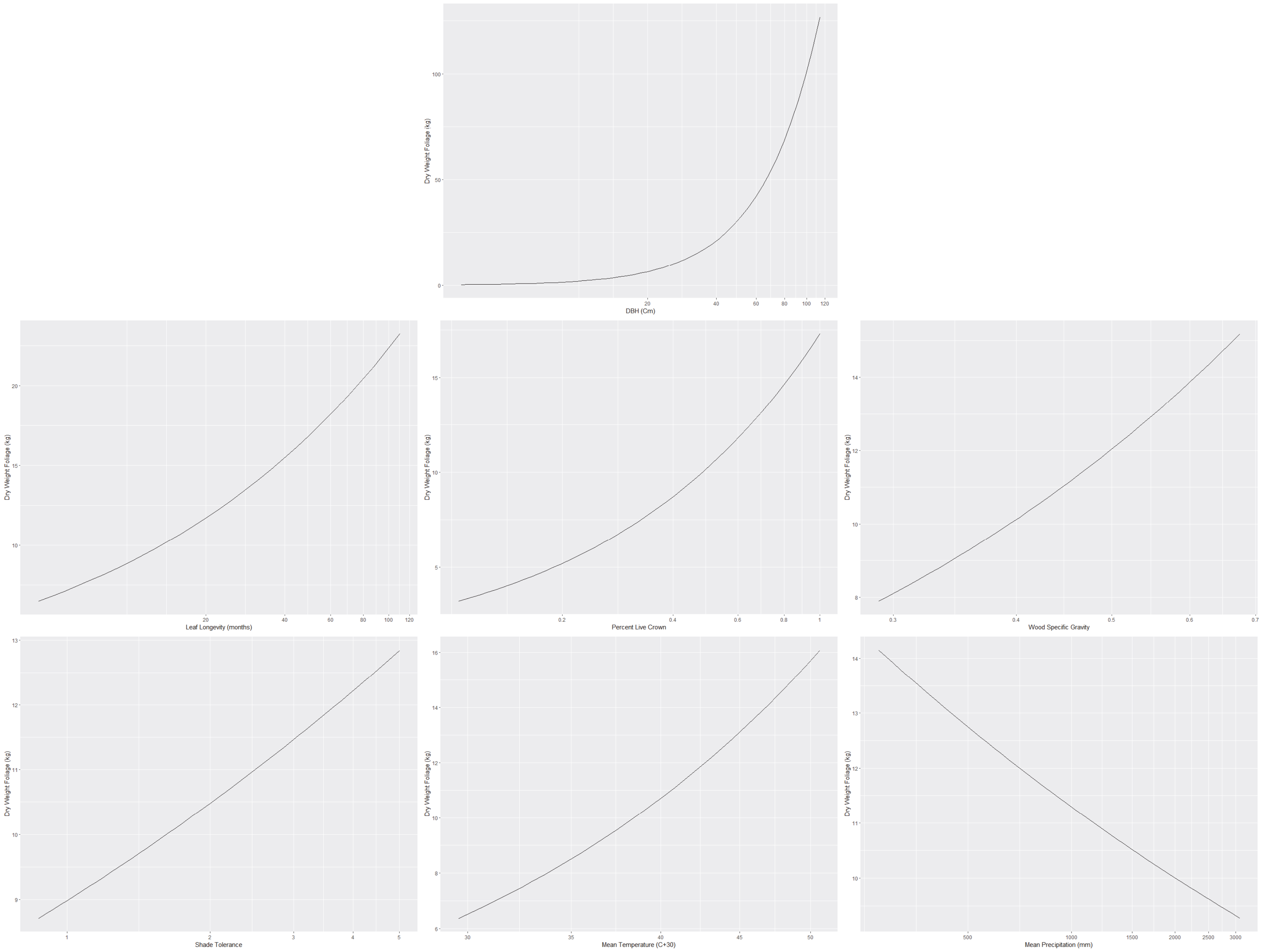

Supplement: Supplementary file 1 — Appendix S1 [file EAP-32-e2646-s001.pdf]
